# 6-(1*H*-Indol-3-yl)-4-phenyl-2,2′-bi­pyridine-5-carbonitrile

**DOI:** 10.1107/S1600536809016195

**Published:** 2009-05-07

**Authors:** Weijun Zhu, Yan Xiang, Songlei Zhu

**Affiliations:** a Xuzhou College of Industrial Technology, Xuzhou 221140, People’s Republic of China; b Xuzhou Pharmaceutical College, Xuzhou 221116, People’s Republic of China; c Xuzhou Medical College, Xuzhou 221002, People’s Republic of China

## Abstract

In the mol­ecule of the title compound, C_25_H_16_N_4_, the pyridine rings are oriented at a dihedral angle of 0.92 (3)°, while the dihedral angle between the benzene ring and the adjacent pyridine ring is 56.51 (3)°. In the crystal structure, inter­molecular N—H⋯N hydrogen bonds link the mol­ecules into centrosymmetric dimers, forming *R*_2_^2^(16) ring motifs. π–π contacts between the pyridine ring and the indole ring system and between the pyridine rings [centroid–centroid distances = 3.923 (2) and 3.724 (2) Å] may further stabilize the structure. Two weak C—H⋯π inter­actions are also present.

## Related literature

For general background, see: da Silva *et al.* (2001 ▶); Joshi & Chand (1982 ▶); Namba *et al.* (2005 ▶). For a related structure, see: Zhu *et al.*, (2008 ▶). For bond-length data, see: Allen *et al.* (1987 ▶). For ring-motifs, see: Bernstein *et al.* (1995 ▶).
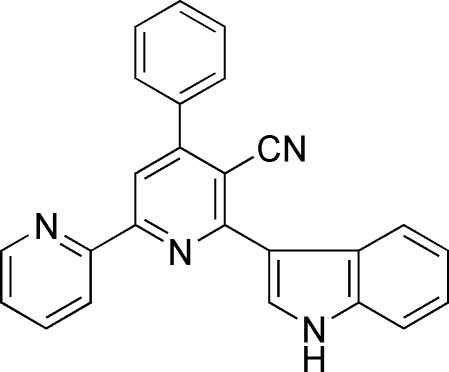


## Experimental

### 

#### Crystal data

C_25_H_16_N_4_
*M* *_r_* = 372.42
Triclinic, 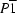

*a* = 9.7744 (16) Å
*b* = 9.7927 (11) Å
*c* = 11.233 (2) Å
α = 73.121 (13)°
β = 86.008 (16)°
γ = 63.853 (10)°
*V* = 921.5 (3) Å^3^
*Z* = 2
Mo *K*α radiation
μ = 0.08 mm^−1^
*T* = 291 K
0.55 × 0.35 × 0.30 mm

#### Data collection

Rigaku Mercury diffractometer
Absorption correction: multi-scan (*ABSCOR*; Jacobson, 1998 ▶) *T*_min_ = 0.966, *T*_max_ = 0.976
8987 measured reflections
3349 independent reflections
2614 reflections with *I* > 2/s(*I*)
*R*_int_ = 0.027

#### Refinement

*R*[*F* ^2^ > 2σ(*F* ^2^)] = 0.054
*wR*(*F* ^2^) = 0.126
*S* = 1.12
3349 reflections
263 parameters
H-atom parameters constrained
Δρ_max_ = 0.16 e Å^−3^
Δρ_min_ = −0.21 e Å^−3^

### 

Data collection: *CrystalClear* (Rigaku/MSC, 2001 ▶); cell refinement: *CrystalClear*; data reduction: *CrystalStructure* (Rigaku/MSC, 2004 ▶); program(s) used to solve structure: *SHELXS97* (Sheldrick, 2008 ▶); program(s) used to refine structure: *SHELXL97* (Sheldrick, 2008 ▶); molecular graphics: *ORTEPII* (Johnson, 1976 ▶) and *PLATON* (Spek, 2009 ▶); software used to prepare material for publication: *SHELXL97* and *PLATON*.

## Supplementary Material

Crystal structure: contains datablocks global, I. DOI: 10.1107/S1600536809016195/hk2679sup1.cif

Structure factors: contains datablocks I. DOI: 10.1107/S1600536809016195/hk2679Isup2.hkl

Additional supplementary materials:  crystallographic information; 3D view; checkCIF report

## Figures and Tables

**Table 1:** Hydrogen-bond geometry (Å, °)

*D*—H⋯*A*	*D*—H	H⋯*A*	*D*⋯*A*	*D*—H⋯*A*
N4—H4⋯N3^i^	0.86	2.23	3.066 (2)	164
C12—H12⋯*Cg*5^ii^	0.93	2.84	3.649 (3)	146
C23—H23⋯*Cg*4^iii^	0.93	2.91	3.711 (3)	145

Symmetry codes: (i) 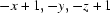; (ii) 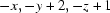; (iii) 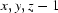. *Cg*4 and *Cg*5 are the centroids of the C11–C16 and C20–C25 rings, respectively.

**Fig. 1.**
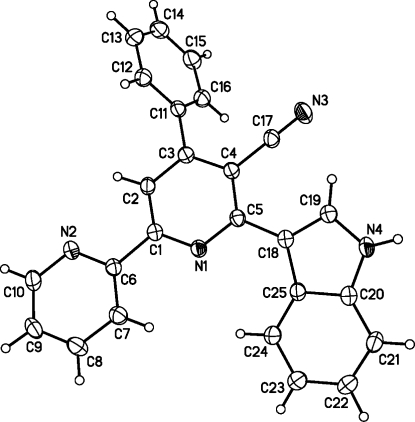
The molecular structure of the title molecule, with the atom-numbering scheme. Displacement ellipsoids are drawn at the 30% probability level.

**Fig. 2.**
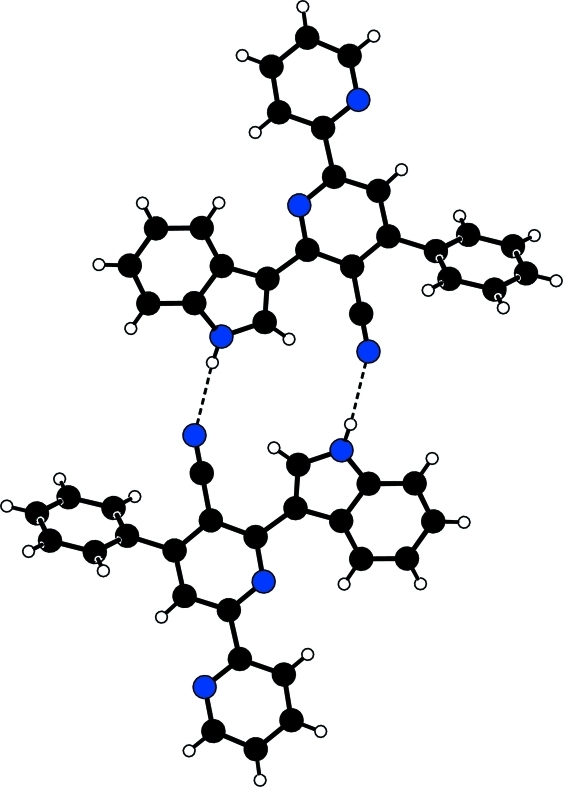
A partial packing diagram of the title compound. Hydrogen bonds are shown as dashed lines.

## supplementary crystallographic information

### Comment

Indole nucleus is a well known heterocycle (da Silva *et al.*, 2001). Compounds carrying the indole moiety exhibit antibacterial and fungicidal activities (Joshi & Chand, 1982). Moreover, the bipyridines and the related complexes have also found numerous applications in asymmetric catalysis, photoinduced electron transfer, and polymer and dendrimer science (Namba *et al.*, 2005). As a part of our programme devoted to the preparation of functionalized indole derivatives, we synthesized a series of indole substituted heterocycles (Zhu *et al.*,  2008). We report herein the crystal structure of the title compound.

In the molecule of the title compound (Fig 1), the bond lengths (Allen *et al.*,  1987) and angles are within normal ranges. The indole ring system A (N4/C18-C25) is planar with a maximum deviation of -0.021 (3) Å for atom C25. Rings B (N1/C1-C5), C (N2/C6-C10) and D (C11-C16) are, of course, planar, and they are oriented at dihedral angles of A/B = 23.01 (3), A/C = 23.61 (3), A/D = 74.90 (3), B/C = 0.92 (3), B/D = 56.51 (3) and C/D = 56.51 (3) °. So, rings B and C are nearly coplanar.

In the crystal structure, intermolecular N-H···N hydrogen bonds (Table 1) link the molecules into centrosymmetric dimers forming R_2_^2^(16) ring motifs (Fig. 2) (Bernstein *et al.*, 1995), in which they may be effective in the stabilization of the structure. The π–π contacts between the pyridine ring and the indole ring system and the pyridine rings, Cg1—Cg2^i^ and Cg2—Cg3^ii^ [symmetry codes: (i) -x, 2 - y, 1 - z, (ii) -x, 1 - y, 1 - z, where Cg1, Cg2 and Cg3 are centroids of the rings (N4/C18-C20/C25), B (N1/C1-C5) and C (N2/C6-C10), respectively] may further stabilize the structure, with centroid-centroid distances of 3.923 (2) and 3.724 (2) Å. There also exist two weak C—H···π interactions (Table 1).

### Experimental

The title compound was prepared by one-pot reaction of 3-cyanoacetyl indole (2 mmol), benzaldehyde (2 mmol) and  2-acetyl pyridine (2 mmol) in present of ammonium acetate in ethanol. After refluxing for 5 h, the reaction mixture was cooled and washed with small amount of cool ethanol. The crude product was filtered and single crystals of the title compound were obtained from ethanol solution by slow evaporation at room temperature (yield; 80%, m.p. 567-568 K). Spectroscopic analysis: IR (KBr, n, cm^-1^): 3337, 3050, 2218, 1573, 1535, 1438, 1214, 1145, 850, 745, 703. ^1^H NMR (400 MHz, DMSO-d_6_): 11.89 (br s, 1H, NH), 8.78 (d, J = 4.4 Hz, 1H, ArH), 8.58 (d, J = 8.0 Hz, 1H, ArH), 8.41 (d, J = 5.2 Hz, 2H, ArH), 8.31 (s, 1H, ArH), 8.11 (m, 1H, ArH), 7.79-7.81 (m, 2H, ArH), 7.56-7.66 (m, 5H, ArH), 7.24-7.29 (m, 2H, ArH)

### Refinement

H atoms were positioned geometrically, with N-H = 0.86 Å (for NH) and C-H = 0.93 Å for aromatic H and constrained to ride on their parent atoms with U_iso_(H) = 1.2U_eq_(C,N).

### Crystal data

**Table:**

C_25_H_16_N_4_	*Z* = 2
*M**_r_* = 372.42	*F*(000) = 388
Triclinic, *P*1	*D*_x_ = 1.342 Mg m^−^^3^
Hall symbol: -P 1	Melting point = 567–568 K
*a* = 9.7744 (16) Å	Mo *K*α radiation, λ = 0.71070 Å
*b* = 9.7927 (11) Å	Cell parameters from 3008 reflections
*c* = 11.233 (2) Å	θ = 3.1–25.3°
α = 73.121 (13)°	µ = 0.08 mm^−^^1^
β = 86.008 (16)°	*T* = 291 K
γ = 63.853 (10)°	Block, yellow
*V* = 921.5 (3) Å^3^	0.55 × 0.35 × 0.30 mm

### Data collection

**Table:**

Rigaku Mercury diffractometer	3349 independent reflections
Radiation source: fine-focus sealed tube	2614 reflections with *I* > 2/s(*I*)
graphite	*R*_int_ = 0.027
Detector resolution: 7.31 pixels mm^-1^	θ_max_ = 25.3°, θ_min_ = 3.1°
ω scans	*h* = −11→11
Absorption correction: multi-scan (*ABSCOR*; Jacobson, 1998)	*k* = −10→11
*T*_min_ = 0.966, *T*_max_ = 0.976	*l* = −13→13
8987 measured reflections	

### Refinement

**Table:**

Refinement on *F*^2^	Primary atom site location: structure-invariant direct methods
Least-squares matrix: full	Secondary atom site location: difference Fourier map
*R*[*F*^2^ > 2σ(*F*^2^)] = 0.054	Hydrogen site location: inferred from neighbouring sites
*wR*(*F*^2^) = 0.126	H-atom parameters constrained
*S* = 1.12	*w* = 1/[σ^2^(*F*_o_^2^) + (0.0538*P*)^2^ + 0.1499*P*] where *P* = (*F*_o_^2^ + 2*F*_c_^2^)/3
3349 reflections	(Δ/σ)_max_ < 0.001
263 parameters	Δρ_max_ = 0.16 e Å^−^^3^
0 restraints	Δρ_min_ = −0.20 e Å^−^^3^

### Special details

**Table:**

Geometry. All esds (except the esd in the dihedral angle between two l.s. planes) are estimated using the full covariance matrix. The cell esds are taken into account individually in the estimation of esds in distances, angles and torsion angles; correlations between esds in cell parameters are only used when they are defined by crystal symmetry. An approximate (isotropic) treatment of cell esds is used for estimating esds involving l.s. planes.
Refinement. Refinement of F^2^ against ALL reflections. The weighted R-factor wR and goodness of fit S are based on F^2^, conventional R-factors R are based on F, with F set to zero for negative F^2^. The threshold expression of F^2^ > 2sigma(F^2^) is used only for calculating R-factors(gt) etc. and is not relevant to the choice of reflections for refinement. R-factors based on F^2^ are statistically about twice as large as those based on F, and R- factors based on ALL data will be even larger.

### Fractional atomic coordinates and isotropic or equivalent isotropic displacement parameters (Å^2^)

**Table:**

	*x*	*y*	*z*	*U*_iso_*/*U*_eq_	
N1	0.94201 (16)	0.20772 (17)	0.54741 (13)	0.0355 (4)	
N2	1.20413 (18)	0.3774 (2)	0.44026 (14)	0.0455 (4)	
N3	0.5855 (2)	0.1895 (2)	0.28547 (16)	0.0606 (5)	
N4	0.64100 (17)	−0.04598 (18)	0.69177 (14)	0.0422 (4)	
H4	0.5936	−0.1043	0.7025	0.051*	
C1	1.01436 (19)	0.2799 (2)	0.46717 (16)	0.0347 (4)	
C2	0.98275 (19)	0.3310 (2)	0.33960 (16)	0.0371 (4)	
H2	1.0382	0.3776	0.2873	0.045*	
C3	0.86846 (19)	0.3126 (2)	0.28960 (16)	0.0353 (4)	
C4	0.79281 (19)	0.2362 (2)	0.37308 (16)	0.0348 (4)	
C5	0.83308 (19)	0.1829 (2)	0.50305 (16)	0.0334 (4)	
C6	1.13427 (19)	0.3032 (2)	0.52184 (16)	0.0354 (4)	
C7	1.1727 (2)	0.2494 (2)	0.64913 (18)	0.0449 (5)	
H7	1.1226	0.1981	0.7039	0.054*	
C8	1.2862 (2)	0.2731 (3)	0.6929 (2)	0.0519 (5)	
H8	1.3135	0.2383	0.7779	0.062*	
C9	1.3586 (2)	0.3482 (2)	0.6104 (2)	0.0494 (5)	
H9	1.4361	0.3648	0.6378	0.059*	
C10	1.3136 (2)	0.3981 (3)	0.4861 (2)	0.0521 (5)	
H10	1.3625	0.4497	0.4302	0.063*	
C11	0.8284 (2)	0.3777 (2)	0.15322 (16)	0.0379 (4)	
C12	0.9407 (2)	0.3356 (2)	0.07066 (17)	0.0445 (5)	
H12	1.0400	0.2617	0.1010	0.053*	
C13	0.9065 (3)	0.4026 (3)	−0.05660 (18)	0.0529 (6)	
H13	0.9823	0.3725	−0.1114	0.063*	
C14	0.7602 (3)	0.5140 (3)	−0.10229 (19)	0.0555 (6)	
H14	0.7376	0.5599	−0.1878	0.067*	
C15	0.6484 (3)	0.5570 (3)	−0.02151 (19)	0.0532 (6)	
H15	0.5498	0.6323	−0.0525	0.064*	
C16	0.6809 (2)	0.4891 (2)	0.10587 (18)	0.0460 (5)	
H16	0.6039	0.5181	0.1600	0.055*	
C17	0.6772 (2)	0.2108 (2)	0.32443 (17)	0.0423 (5)	
C18	0.7603 (2)	0.1025 (2)	0.59690 (16)	0.0352 (4)	
C19	0.6856 (2)	0.0176 (2)	0.58127 (17)	0.0385 (4)	
H19	0.6682	0.0056	0.5054	0.046*	
C20	0.6833 (2)	−0.0028 (2)	0.78422 (17)	0.0380 (4)	
C21	0.6582 (2)	−0.0376 (2)	0.90989 (18)	0.0476 (5)	
H21	0.6081	−0.1001	0.9444	0.057*	
C22	0.7105 (3)	0.0240 (3)	0.98141 (19)	0.0568 (6)	
H22	0.6957	0.0029	1.0663	0.068*	
C23	0.7851 (3)	0.1175 (3)	0.92946 (19)	0.0601 (6)	
H23	0.8188	0.1581	0.9803	0.072*	
C24	0.8104 (2)	0.1515 (3)	0.80452 (18)	0.0507 (5)	
H24	0.8605	0.2143	0.7711	0.061*	
C25	0.7595 (2)	0.0901 (2)	0.72879 (16)	0.0366 (4)	

### Atomic displacement parameters (Å^2^)

**Table:**

	*U*^11^	*U*^22^	*U*^33^	*U*^12^	*U*^13^	*U*^23^
N1	0.0353 (8)	0.0367 (9)	0.0374 (8)	−0.0191 (7)	0.0027 (7)	−0.0097 (7)
N2	0.0430 (9)	0.0583 (11)	0.0475 (9)	−0.0321 (8)	0.0058 (8)	−0.0173 (8)
N3	0.0675 (12)	0.0748 (13)	0.0507 (10)	−0.0502 (11)	−0.0130 (9)	0.0006 (9)
N4	0.0439 (9)	0.0449 (9)	0.0476 (9)	−0.0298 (8)	0.0052 (7)	−0.0109 (7)
C1	0.0320 (9)	0.0349 (10)	0.0378 (10)	−0.0153 (8)	0.0024 (8)	−0.0102 (8)
C2	0.0336 (9)	0.0415 (11)	0.0369 (10)	−0.0206 (8)	0.0024 (8)	−0.0058 (8)
C3	0.0354 (10)	0.0346 (10)	0.0355 (10)	−0.0159 (8)	0.0005 (8)	−0.0081 (8)
C4	0.0346 (9)	0.0346 (10)	0.0362 (10)	−0.0172 (8)	0.0001 (8)	−0.0082 (8)
C5	0.0329 (9)	0.0314 (9)	0.0385 (10)	−0.0157 (8)	0.0034 (8)	−0.0114 (8)
C6	0.0319 (9)	0.0355 (10)	0.0407 (10)	−0.0152 (8)	0.0036 (8)	−0.0134 (8)
C7	0.0452 (11)	0.0490 (12)	0.0415 (11)	−0.0237 (10)	−0.0025 (9)	−0.0083 (9)
C8	0.0514 (12)	0.0575 (13)	0.0492 (12)	−0.0256 (11)	−0.0101 (10)	−0.0131 (10)
C9	0.0334 (10)	0.0567 (13)	0.0663 (14)	−0.0199 (10)	−0.0016 (10)	−0.0281 (11)
C10	0.0458 (12)	0.0661 (14)	0.0611 (13)	−0.0364 (11)	0.0121 (10)	−0.0249 (11)
C11	0.0418 (10)	0.0419 (11)	0.0360 (10)	−0.0255 (9)	0.0003 (8)	−0.0080 (8)
C12	0.0459 (11)	0.0509 (12)	0.0399 (11)	−0.0266 (10)	0.0016 (9)	−0.0089 (9)
C13	0.0668 (14)	0.0664 (15)	0.0398 (11)	−0.0420 (13)	0.0114 (10)	−0.0171 (10)
C14	0.0770 (16)	0.0650 (15)	0.0357 (11)	−0.0472 (13)	−0.0048 (11)	−0.0026 (10)
C15	0.0566 (13)	0.0545 (13)	0.0463 (12)	−0.0306 (11)	−0.0118 (11)	0.0018 (10)
C16	0.0448 (11)	0.0539 (12)	0.0410 (10)	−0.0260 (10)	−0.0005 (9)	−0.0085 (9)
C17	0.0457 (11)	0.0469 (12)	0.0382 (10)	−0.0283 (10)	0.0001 (9)	−0.0042 (9)
C18	0.0334 (9)	0.0345 (10)	0.0388 (10)	−0.0164 (8)	0.0030 (8)	−0.0098 (8)
C19	0.0408 (10)	0.0398 (11)	0.0387 (10)	−0.0215 (9)	0.0030 (8)	−0.0109 (8)
C20	0.0339 (10)	0.0373 (10)	0.0436 (11)	−0.0174 (8)	0.0041 (8)	−0.0105 (8)
C21	0.0476 (12)	0.0545 (13)	0.0449 (11)	−0.0305 (10)	0.0111 (9)	−0.0091 (10)
C22	0.0709 (15)	0.0729 (15)	0.0377 (11)	−0.0436 (13)	0.0129 (10)	−0.0146 (11)
C23	0.0815 (16)	0.0805 (16)	0.0432 (12)	−0.0570 (14)	0.0122 (11)	−0.0205 (11)
C24	0.0656 (14)	0.0626 (13)	0.0424 (11)	−0.0457 (12)	0.0096 (10)	−0.0145 (10)
C25	0.0356 (10)	0.0374 (10)	0.0394 (10)	−0.0199 (8)	0.0033 (8)	−0.0091 (8)

### Geometric parameters (Å, °)

**Table:**

N1—C1	1.339 (2)	C11—C12	1.385 (3)
N1—C5	1.347 (2)	C11—C16	1.391 (3)
N2—C10	1.334 (2)	C12—C13	1.384 (3)
N2—C6	1.341 (2)	C12—H12	0.9300
N3—C17	1.144 (2)	C13—C14	1.378 (3)
N4—C19	1.354 (2)	C13—H13	0.9300
N4—C20	1.377 (2)	C14—C15	1.369 (3)
N4—H4	0.8600	C14—H14	0.9300
C1—C2	1.381 (2)	C15—C16	1.384 (3)
C1—C6	1.490 (2)	C15—H15	0.9300
C2—C3	1.385 (2)	C16—H16	0.9300
C2—H2	0.9300	C18—C19	1.375 (2)
C3—C4	1.404 (2)	C18—C25	1.451 (2)
C3—C11	1.484 (2)	C19—H19	0.9300
C4—C5	1.420 (2)	C20—C21	1.384 (3)
C4—C17	1.432 (2)	C20—C25	1.405 (2)
C5—C18	1.465 (2)	C21—C22	1.372 (3)
C6—C7	1.388 (2)	C21—H21	0.9300
C7—C8	1.377 (3)	C22—C23	1.389 (3)
C7—H7	0.9300	C22—H22	0.9300
C8—C9	1.368 (3)	C23—C24	1.376 (3)
C8—H8	0.9300	C23—H23	0.9300
C9—C10	1.371 (3)	C24—C25	1.398 (3)
C9—H9	0.9300	C24—H24	0.9300
C10—H10	0.9300		
			
C1—N1—C5	119.18 (15)	C13—C12—H12	119.7
C10—N2—C6	117.30 (17)	C11—C12—H12	119.7
C19—N4—C20	109.41 (15)	C14—C13—C12	120.1 (2)
C19—N4—H4	125.3	C14—C13—H13	119.9
C20—N4—H4	125.3	C12—C13—H13	119.9
N1—C1—C2	123.09 (15)	C15—C14—C13	119.85 (19)
N1—C1—C6	116.67 (15)	C15—C14—H14	120.1
C2—C1—C6	120.24 (16)	C13—C14—H14	120.1
C1—C2—C3	119.94 (16)	C14—C15—C16	120.5 (2)
C1—C2—H2	120.0	C14—C15—H15	119.8
C3—C2—H2	120.0	C16—C15—H15	119.8
C2—C3—C4	117.25 (16)	C15—C16—C11	120.2 (2)
C2—C3—C11	119.47 (16)	C15—C16—H16	119.9
C4—C3—C11	123.25 (15)	C11—C16—H16	119.9
C3—C4—C5	120.12 (15)	N3—C17—C4	179.5 (2)
C3—C4—C17	118.82 (15)	C19—C18—C25	105.64 (15)
C5—C4—C17	121.05 (16)	C19—C18—C5	128.12 (16)
N1—C5—C4	120.37 (15)	C25—C18—C5	126.18 (15)
N1—C5—C18	115.71 (15)	N4—C19—C18	110.50 (16)
C4—C5—C18	123.91 (15)	N4—C19—H19	124.7
N2—C6—C7	122.15 (16)	C18—C19—H19	124.7
N2—C6—C1	115.83 (15)	N4—C20—C21	129.29 (17)
C7—C6—C1	122.01 (16)	N4—C20—C25	107.56 (15)
C8—C7—C6	118.82 (18)	C21—C20—C25	123.15 (17)
C8—C7—H7	120.6	C22—C21—C20	117.15 (18)
C6—C7—H7	120.6	C22—C21—H21	121.4
C9—C8—C7	119.50 (19)	C20—C21—H21	121.4
C9—C8—H8	120.2	C21—C22—C23	121.23 (19)
C7—C8—H8	120.2	C21—C22—H22	119.4
C8—C9—C10	118.03 (18)	C23—C22—H22	119.4
C8—C9—H9	121.0	C24—C23—C22	121.5 (2)
C10—C9—H9	121.0	C24—C23—H23	119.2
N2—C10—C9	124.20 (19)	C22—C23—H23	119.2
N2—C10—H10	117.9	C23—C24—C25	118.88 (18)
C9—C10—H10	117.9	C23—C24—H24	120.6
C12—C11—C16	118.78 (17)	C25—C24—H24	120.6
C12—C11—C3	119.99 (16)	C24—C25—C20	118.06 (17)
C16—C11—C3	121.11 (17)	C24—C25—C18	135.01 (17)
C13—C12—C11	120.51 (19)	C20—C25—C18	106.88 (15)
			
C5—N1—C1—C2	0.0 (3)	C16—C11—C12—C13	−0.3 (3)
C5—N1—C1—C6	−179.68 (15)	C3—C11—C12—C13	−176.21 (17)
N1—C1—C2—C3	2.0 (3)	C11—C12—C13—C14	1.0 (3)
C6—C1—C2—C3	−178.34 (16)	C12—C13—C14—C15	−0.7 (3)
C1—C2—C3—C4	−2.2 (3)	C13—C14—C15—C16	−0.1 (3)
C1—C2—C3—C11	175.87 (17)	C14—C15—C16—C11	0.8 (3)
C2—C3—C4—C5	0.7 (3)	C12—C11—C16—C15	−0.5 (3)
C11—C3—C4—C5	−177.30 (16)	C3—C11—C16—C15	175.28 (17)
C2—C3—C4—C17	−178.14 (16)	N1—C5—C18—C19	−157.15 (17)
C11—C3—C4—C17	3.8 (3)	C4—C5—C18—C19	24.1 (3)
C1—N1—C5—C4	−1.6 (2)	N1—C5—C18—C25	19.5 (3)
C1—N1—C5—C18	179.67 (15)	C4—C5—C18—C25	−159.21 (17)
C3—C4—C5—N1	1.2 (3)	C20—N4—C19—C18	0.8 (2)
C17—C4—C5—N1	−179.95 (17)	C25—C18—C19—N4	−0.5 (2)
C3—C4—C5—C18	179.84 (16)	C5—C18—C19—N4	176.68 (17)
C17—C4—C5—C18	−1.3 (3)	C19—N4—C20—C21	178.41 (19)
C10—N2—C6—C7	0.0 (3)	C19—N4—C20—C25	−0.8 (2)
C10—N2—C6—C1	−179.08 (16)	N4—C20—C21—C22	−178.64 (19)
N1—C1—C6—N2	−179.21 (15)	C25—C20—C21—C22	0.5 (3)
C2—C1—C6—N2	1.1 (3)	C20—C21—C22—C23	0.1 (3)
N1—C1—C6—C7	1.7 (3)	C21—C22—C23—C24	−0.3 (4)
C2—C1—C6—C7	−178.01 (17)	C22—C23—C24—C25	0.0 (4)
N2—C6—C7—C8	0.0 (3)	C23—C24—C25—C20	0.6 (3)
C1—C6—C7—C8	179.07 (17)	C23—C24—C25—C18	177.8 (2)
C6—C7—C8—C9	−0.3 (3)	N4—C20—C25—C24	178.46 (16)
C7—C8—C9—C10	0.4 (3)	C21—C20—C25—C24	−0.8 (3)
C6—N2—C10—C9	0.2 (3)	N4—C20—C25—C18	0.5 (2)
C8—C9—C10—N2	−0.4 (3)	C21—C20—C25—C18	−178.79 (17)
C2—C3—C11—C12	54.4 (2)	C19—C18—C25—C24	−177.5 (2)
C4—C3—C11—C12	−127.63 (19)	C5—C18—C25—C24	5.3 (3)
C2—C3—C11—C16	−121.39 (19)	C19—C18—C25—C20	0.0 (2)
C4—C3—C11—C16	56.6 (3)	C5—C18—C25—C20	−177.27 (16)

### Hydrogen-bond geometry (Å, °)

**Table:**

*D*—H···*A*	*D*—H	H···*A*	*D*···*A*	*D*—H···*A*
N4—H4···N3^i^	0.86	2.23	3.066 (2)	164
C12—H12···Cg5^ii^	0.93	2.84	3.649 (3)	146
C23—H23···Cg4^iii^	0.93	2.91	3.711 (3)	145

Symmetry codes: (i) −*x*+1, −*y*, −*z*+1; (ii) −*x*, −*y*+2, −*z*+1; (iii) *x*, *y*, *z*−1.
